# Cross-cultural effects of reminiscence therapy on life satisfaction and autobiographical memory of older adults: a pilot study across Mexico and Spain

**DOI:** 10.1186/s13195-023-01347-x

**Published:** 2023-11-22

**Authors:** Alba Villasán Rueda, Antonio Sánchez Cabaco, Manuel Alejandro Mejía-Ramírez, Rosa Marina Afonso, Eduardo Castillo-Riedel

**Affiliations:** 1 Faculty of Health Sciences, Catholic University of Ávila, Calle de los Canteros, s/n, 05005 Ávila, Spain; 2 Faculty of Psychology, Pontifical University of Salamanca, 37002 Salamanca, Spain; 3 School of Psychology, CETYS University, Campus Tijuana, 22210 Tijuana, Baja California Mexico; 4 Department of Psychology and Education, University of Beira Interior, 6201-001 Covilhã, Portugal

**Keywords:** Aging, Cognitive stimulation, Quality of life, Reminiscence, Specific memory

## Abstract

**Background:**

There are increasing reports on the cognitive and emotional benefits of positive reminiscence therapy in older people. The objective of this study is to assess the differential improvement of the quality of life for older people in different vital situations (three different types of aging) and from different countries by implementing a positive reminiscence therapy program (REMPOS).

**Methods:**

The participants were 144 older adults above the age of 65, 77 participants from Spain (45 experimental groups, 32 control groups) and 67 from Mexico (34 experimental groups, 33 control groups). The participants were recruited from nursing and retirement homes. A factorial randomized design with pre–post measurement with three independent variables: country (Mexico, Spain), condition (experimental, control), and types of aging (healthy aging, HA., mild cognitive impairment, MCI., Alzheimer’s disease, AD). The experimental groups received REMPOS therapy and control groups received standard cognitive stimulation program. The quality of life was measured with the Life Satisfaction Inventory for adults (LSI-A) and autobiographical memory test (AMT) before and after REMPOS therapy.

**Results:**

The REMPOS intervention showed significantly higher positive effects than the control condition on the recall of specific positive memories across countries and types of aging, except for the Spanish MCI group. Life satisfaction in the Alzheimer’s and MCI group only improved with REMPOS in the Mexican sample.

**Conclusions:**

The REMPOS effects showed generalizable effects across countries, but the cross-cultural differences shown highlight the necessity of running studies to test those differential effects.

**Supplementary Information:**

The online version contains supplementary material available at 10.1186/s13195-023-01347-x.

## Introduction

The current situation derived from the mental health effects of the COVID-19 pandemic, the increase in problems of loneliness in the elderly, the social distancing, or the institutionalization itself in older adults living in Residential Care Facilities (RCFs) [1, 2] increase the psychosocial and cognitive risk. It is crucial to focus on the vulnerability of the older adults, especially in preventing memory dysfunction since affective disorders decrease the ability to recall specific memories, something that is currently on the rise in the older population. [3] Williams et al. [4] explain through the CaR-FA-X theoretical model that an underlying overgeneralization of memories causes the decrease in the ability to recall specific memories. This model states that this phenomenon derives from three mechanisms: capture and rumination (an excessive activation of emotional representation related to oneself), functional avoidance (passively avoiding aversive memories), and impaired executive memory (limited processing capacity).

Older adults with depression tend to recall more negative past experiences than positive, this in turn creates a negative bias that reinforces and increases the current emotional lability [5], which also negatively impacts working memory [3, 6]. Some studies suggest that people with depression showed less positive memory recall and less specific memory recall in an autobiographical memory test (AMT) than healthy subjects [7]. In addition, the valence of these negative memories intensified for depressed older people; this is why it is imperative to intervene and modify the cognitive-emotional variables that cause and maintain these overgeneralized memories (OGM) which lean towards negative memories. [8].

Recent studies show that reminiscence therapy helps improve autobiographical memories, also making it possible to rewrite personal experiences [9, 10]. Reminiscence therapy is mainly used to treat cognitive and emotional problems in older people [11–15], besides not having secondary adverse effects as pharmacological therapies. There is little to no literature on the comparison of the continuum in the aging process from a normal to a pathological aging, using the same therapeutic interventions and evaluating similar dimensions. Moreover, we refer specifically to the efficacy in the transition stage between both realities, which is the clinical entity of mild cognitive impairment [16, 17].

Among the different types of reminiscence programs, REMPOS was developed by Cabaco [18]. In this program, generic or specific triggering stimuli are used to facilitate accessibility to autobiographical memories, specifically to positive memories. REMPOS was developed based on the Review of Life based on Specific Positive Events [19], converting the intervention into a group program with a broader content structure. In the REMPOS program, the amount of sessions and objectives were extended, but both programs share the epistemological foundation in Positive Psychology [20]. REMPOS has shown effectiveness in improving cognitive and emotional variables of healthy and impaired older people [21, 22]. In a preliminary cross-cultural study [23], which examined the effects of the REMPOS program on cognitive and emotional factors among elderly individuals in Mexico and Spain, the findings were exceptionally positive. The intervention yielded significant improvements in cognitive function and a reduction in depressive symptoms in both healthy older adults and those with early cognitive impairment in both countries. It is noteworthy to emphasize that the intervention had a more pronounced positive impact on cognitive and emotional aspects in subgroups with cognitive impairment, specifically in individuals with incipient Alzheimer’s disease. The REMPOS program, as part of the studies mentioned [21–23], demonstrated a significant reduction in depressive symptoms, enhancements in cognition, and an increase in life satisfaction—crucial components of the psychological well-being of older individuals. Furthermore, it has been incorporated into a comprehensive intervention program, considering its associations with other forms of reserves (cognitive, physical, and motivational), to mitigate the risk of deterioration and promote healthy aging [24, 25].

There are cultural differences between Mexico and Spain in the understanding of quality of life and the components associated with it. Thus, in Spain, external elements such as social relations and satisfaction with the environment are considered more relevant for quality of life, while for Mexico internal elements, such as health and functional capacity, are more important [26]. Similarly, data from the Organization for Economic Cooperation and Development (OECD) shows that Mexico is one of the countries with the highest social loneliness since 21.3% of older people was classified as lacking social support, meaning that they reported having no friends or relatives to count on in times of trouble, while only 8.0% of older adults in Spain were classified as lacking social support [27]. Life satisfaction, on the other hand, has been reported to be more similar between Mexico and Spain, both countries having scores near the general average of the OECD countries [28].

In addition, it has been proposed that culture influences the relationship between personality and life satisfaction, a relationship that is also mediated by affective balance. For example, a study compared samples from individualistic and collectivist cultures [29], observing that the influence of personality on affective balance is not modified by culture but the impact of emotional balance on life satisfaction, this being more important for individualistic cultures [30]. For this reason, the emotional value given to different situations in daily life and how this influences happiness is determined by the cultural context. Taking this into account, the same could happen with situations such as loneliness, since its interpretation could be determined by the culture of the older person who is alone. In the case of this study, Spain has a more individualistic culture than Mexico, so the subjective perception of loneliness could be different for both countries [29].

This study aims to analyze the cross-cultural differences in the efficacy of the REMPOS therapy program in comparison to a standard classic intervention (cognitive stimulation) in terms of life satisfaction and autobiographical memory. In addition, a secondary aim is to verify the differential efficacy of the REMPOS program in healthy older people and older people with different levels of deterioration. There are three main hypotheses: H1 There is a cross-cultural difference in the effect of the REMPOS program compared to a control condition on the LSI-A scores; H2 There is a cross-cultural difference in the effect of the REMPOS program compared to a control condition on the EPOS scores; and H3 There is a cross-cultural difference in the effect of the REMPOS program compared to a control condition on the ENEG scores. To gain deeper insights into the analysis, we tested these three hypotheses in three different aging categories, namely healthy aging (HA), mild cognitive impairment (MCI), and Alzheimer’s disease (AD). These hypotheses required three independent variables: condition (experimental and control groups), time of testing (pre- or postintervention), and country, namely Mexico and Spain, constituting the cross-cultural comparison. On the other hand, our dependent variables encompass the test scores, specifically those from the Life Satisfaction Inventory (LSI-A) and the autobiographical memory test (AMT). This design allowed us to measure and compare the effects of both types of therapies in each country across all three different aging categories.

## Method

### Participants and design

The participants were 144 older adults, 77 from Spain and 67 from Mexico. Proportions of each gender were similar in both samples, 75.3% females (58 females, 19 males) in the Spanish sample and 76.1% females (51 females, 16 males) in the Mexican ample, giving a total of 75.7% females (109 females, 35 males) in the full sample. The mean age was 79.9 years (SD = 9.28) for the full sample, 83.1 years (SD = 7.54, range between 65 and 97 years) for Spain, and 76.2 years (SD = 9.74, range between 62 and 97 years) for Mexico. Men in both countries had similar mean ages, with 79.3 years (SD = 7.18) for Spanish men and 77.3 years (SD = 8.65) for Mexican men. With a mean age of 84.3 years (SD = 7.3), Spanish women were older than Mexican women in the sample, who had a mean age of 75.8 years (SD = 10.1).

Inclusion criteria included (1) be 65 years of age or older; (2) healthy aging (HA), mild cognitive impairment (MCI), and Alzheimer’s disease (AD); and (3) who resided or visited a residential care facility or day centers where the intervention was carried out.

### Instruments

All participants performed a pre- and posttest assessment which included the Life Satisfaction Inventory, adult version (LSI-A) [31, 32], and the autobiographical memory test (AMT) [33], for which we only analyzed the recall of specific memories for positive stimuli (EPOS), and for negative stimuli (ENEG). Further details of the assessments are already published in Villasán et al. [22] and Villasán et al. [34].

The Life Satisfaction Index for the Elderly (LSI-A) is a 20-item questionnaire developed to assess the well-being of older individuals. Respondents express their agreement or disagreement with general life statements using "agree," "disagree," or "unsure" responses [32, 35]. Scores on the LSI-A range from 0 to 40 points, with "disagree" responses assigned 0 points, "unsure" responses assigned 1 point, and "agree" responses assigned 2 points [36]. A higher total score on the LSI-A reflects greater well-being. Studies have shown that the LSI-A is reliable, with a coefficient of 0.74 when used with older populations [35].

The autobiographical memory test (AMT) [33] is a tool that assesses an individual’s ability to recall self-related memories within a specified time frame using word cues with positive, negative, or neutral emotions. The test includes various word cues, and participants have 60 s to remember and describe a specific memory related to the cue. Memories are categorized as "specific" if they last less than a day at a particular time and place, "extended" if they span multiple days, or "categorical" if they represent generic recurring events. Failure to recall a memory within the time limit or recalling unrelated information is noted as an "omission." Inter-rater reliability was evaluated in a study, with strong agreement observed for positive and negative word cues, indicated by kappa index values of 0.86 and 0.83, respectively [33]. The study primarily focused on specific positive (EPOS) and specific negative (ENEG) memories.

### Procedures

Participants were recruited from 12 institutions located in the city of Salamanca, Spain, and in Tijuana, Mexico. All participants gave their informed consent to be part of the study, following the Helsinki Declaration and Ethics board approval from the Universidad Pontificia de Salamanca. All participants completed a pretest evaluation, then received an intervention, either REMPOS therapy [18] or a standard cognitive stimulation program [28], and finally, completed a posttest evaluation.

The sample was composed of 12 groups, 6 groups within each country, as shown in Table 1. Within each country, there were two groups of each level of aging: healthy aging (HA), mild cognitive impairment (MCI), and Alzheimer’s disease (AD). Half of the groups received REMPOS therapy and were considered the experimental condition, while the other half received standard cognitive stimulation and constituted the control condition. Groups were randomly assigned to either condition. Participants of the HA and MCI groups did not have a previous diagnosis of AD and were classified from their scores in the Spanish version of the MiniMental Examination Questionnaire (MEC) [37, 38]. The HA participants had no indication of cognitive deficit, while MCI participants had indication of cognitive deficit according to cut-offs established for Mexico (HA, MEC ≥ 24 and MCI, MEC < 24) and Spain (HA, MEC > 25) and MCI (MEC < 25). The participants of the AD group had a previous AD diagnosis, verified by their institution.

**Table 1:** Groups formation in each country sample

Groups	Alzheimer	MCI	HA	Total
Spain	26	24	27	77
Control	6	13	13	32
Experimental	20^a^	11	14	45
Mexico	21	22	24	67
Control	10	10	13	33
Experimental	11	12	11	34
Total	47	46	51	144

*GDS* Geriatric Depression Scale, *MCI* Mild cognitive impairment, *HA* Healthy aging

^a^Spain’s Alzheimer experimental group included 10 people with GDS3 and 10 with GDS4

The experimental and control conditions consisted of 12 sessions of either REMPOS therapy or cognitive stimulation therapy, respectively. In both conditions, sessions lasted 1 h and were imparted twice a week. In order to implement both types of intervention programs to the Mexican population, slight cultural and linguistic modifications had to be made to improve the comprehension of the older people. Groups had a maximum of 13 people for the HA and MCI groups, and a maximum of 11 people for the AD groups.

The REMPOS program [18] consists of the following sessions: (1) Introduction to reminiscence, (2) Everyday things, (3) My present-past-future, (4) Interpersonal relationships, (5) Important dates, (6) Celebrating dates/holidays, (7) Occupations and professions, (8) Games, (9) Remembering loved ones, (10) Music and memories, (11) *Reirpos* (positive emotions through laughter), and (12) Laughing more, living more.

The guide used for cognitive stimulation were exercises that were selected in the program developed by Cabaco [39]. The themes of each session were (1) Cues to improve registry: concentration; (2) Organizing information; (3) Visualization and misattributions; (4) The importance of language; (5) Routes and semantic knowledge; (6) Reading and comprehension, and procedural knowledge; (7) Basic math and arithmetics; (8) Math skill stimulation; (9) Relational memory training I; (10) Relational memory training II; (11) Importance of self-regulation and attention; and (12) Breathing exercises.

Posttest evaluation of all the participants (experimental and control group, in both countries) were done between 3 and 3.5 months after pretest.

### Data analysis

There were two main questions for the intended comparison across countries. First, we tested if there were any differences across the three types of aging between Mexico and Spain. For this question, we compared only the pretest scores across all six measures, for the three types of aging, using *t*-tests controlling for type-1 errors by correcting for multiple comparisons with the Holm method. Effect sizes for *t*-tests are reported using Hedge’s *g*.

Second, we analyzed if the effects of the interventions varied across countries. For this question, we ran linear mixed-effects models with the R packages lme4 [40] and lmerTest [41] within each country and type of aging for the prediction of each dependent variable: LSI-A scores, AMT positive specific memories recall (EPOS), and AMT negative specific memories recall (ENEG). The models were fitted by restricted maximum likelihood, and *t*-tests for each coefficient used Satterthwaite’s method. The formula for each model had as predictors the time (pre- vs postscores), the interaction between time and condition (experimental vs control), and an intercept both as fixed- and random-effects (with participants as the grouping factor). This type of model is equivalent to testing the experimental effect using an analysis of covariance in a randomized controlled study design, where the interaction between time and condition is the effect of interest that quantifies the differential effect of the experimental and control interventions.

Building separate models for each type of aging makes the interpretation easier, and it is justified if there are significant interactions with time and condition. Given that we built separate models for each country, we further tested the same models pooling all participants from both countries where we included the triple interaction between time, condition, and country as a predictor. These pooled models add further evidence of differential effects across countries where those are detectable. Finally, to further clarify the interactions in each country, we ran simple main effects analyses for the predictor time within each group condition in each country, by comparing pre- and postintervention scores with paired sample *t*-tests (tables for these are reported in the Supplementary Material).

Scores were assumed robust enough to analyze them with parametric statistics, based on the assumption analyses reported by the authors [22, 34].

## Results

### Differences across countries in baseline scores

Differences in the preintervention scores across countries are shown in Table 2. In the healthy aging sample, the Mexican sample had higher scores in the LSI-A scores (*p* = 0.040, Hedge’s *g* =  − 0.89) and in the AMT negative specific memories recall (*p* = 0.006, Hedge’s *g* =  − 1.07), compared to the Spanish sample.

**Table 2:** Comparison of pretest scores across countries

	Spain Mean (SD)	Mexico Mean (SD)	*t*	*df*	*p*	Hedge’s *g*
Alzheimer	*n* = 26	*n* = 21				
LSI-A	25.2 (5.7)	20.5 (6.9)	2.50	38.6	.204	0.74
EPOS	2.0 (0.9)	1.8 (1.0)	0.71	41.3	1.00	0.21
ENEG	2.2 (1.1)	2.4 (1.5)	− 0.50	35.3	1.00	− 0.15
MCI	*n* = 24	*n* = 22				
LSI-A	21.6 (5.6)	24.6 (6.2)	− 1.72	42.6	.562	− 0.51
EPOS	1.3 (1.1)	2.0 (1.3)	− 2.00	47.4	.543	− 0.59
ENEG	2.1 (1.2)	2.9 (1.7)	− 1.81	37.4	.562	− 0.54
Healthy aging	*n* = 27	*n* = 24				
LSI-A	20.5 (5.8)	25.7 (5.8)	− 3.17	48.3	*.040*	− 0.89
EPOS	1.5 (0.9)	2.3 (1.0)	− 2.98	47.4	.064	− 0.84
ENEG	2.3 (1.3)	3.6 (1.2)	− 3.82	48.7	*.006*	− 1.07

Hypothesis testing are independent samples Welch *t*-test and *p* values are corrected for multiple comparison with Holm correction

### Cross-cultural differences in the effect of the reminiscence program on LSI-A scores

To test the effects of the REMPOS program on LSI-A scores, we estimated the significance of the interaction between time and condition in the mixed model for each type of aging and country as explained in the “Methods” section above.

For LSI-A scores in the Alzheimer’s sample, the triple interaction between time, condition, and country was significant in the pooled model (*t* =  − 2.82, *p* = 0.007, and see unstandardized coefficients in Table 3), indicating that the REMPOS program had a differential effect in each country. Furthermore, the interaction between time and condition was significant only in the Mexican sample. Simple main effects analysis found that neither of the experimental and control groups in the Spanish sample showed pre–postdifferences in LSI-A scores. On the contrary, in the Mexican sample, the experimental group showed pre–postintervention differences, but not the control group (see Supplementary Table 1). In summary, the reminiscence intervention groups reported higher LSI-A scores after the intervention, the effect was stronger in the Mexican sample, and only there the effect reached statistical significance. Overall, the reminiscence intervention showed stronger effects than the control condition in the Alzheimer’s sample of both countries, with stronger effects in the Mexican sample.

**Table 3:** Unstandardized coefficients of linear mixed models predicting LSI-A scores and autobiographical memory test (AMT) scores for positive (EPOS) and negative (ENEG) specific memories to test the effect of the REMPOS program. The columns for Spain and Mexico show separate models for each sample, where the interaction between time and condition test for the differential effect of the REMPOS program. The column “Both” tests a model pooling all participants from both countries and it uses the interaction between time, condition, and country to test cross-cultural effects

	Spain						Mexico					Both			
	Intercept		Time		Time x condition		Intercept		Time	Time x condition		Time x condition		Time x condition x country	
Alzheimer															
LSI-A	25.14	***	− 1.35		3.61		20.48	***	0.07	8.41	***	8.47	***	− 4.79	**
EPOS	1.96	***	0.18		1.36	**	1.76	***	− 0.26	3.14	***	2.93	***	− 1.19	***
ENEG	2.23	***	− 0.13		0.72		2.24	***	− 0.67	0.51		0.40		0.60	
MCI															
LSI-A	21.58	***	− 1.22		2.22		24.59	***	0.26	2.53		3.80	*	− 2.44	
EPOS	1.29	***	0.32		0.29		2.00	***	< .01	2.76	***	2.95	***	− 2.80	***
ENEG	2.13	***	0.18		0.16		2.91	***	− 0.11	1.21		1.35^a^	**	− 1.24^a^	*
Healthy aging															
LSI-A	20.52	***	1.36		2.8		25.67	***	− 0.12	2.97		3.49		− 2.01	
EPOS	1.52	***	2.90	***	− 0.10		2.29	***	− 0.43	2.91	***	2.87	***	− 2.92	***
ENEG	2.26	***	1.74	***	0.14		3.58	***	− 1.38 ***	2.05	***	2.01^a^	***	− 1.98^a^	**

*p*-values are based on *t*-tests using Satterwhite’s method comparing the coefficients against zero

*MCI* mild cognitive impairment

^a^These models also included the interaction between country and time to avoid singular fits in the variance–covariance matrices

^*^p < .05, **p < .01, ***p < .001

For the LSI-A scores in the MCI sample, the triple interaction in the pooled model between predictors time, condition, and country was not significant (*t* =  − 1.37, *p* = 0.18, see Table 3), but the interaction between time and condition was significant (*t* = 2.49, *p* = 0.016). Unexpectedly, neither sample of each country showed a significant interaction between time and condition, though the coefficient was in the direction of larger LSI-A scores after the intervention. In the simple main effects analyses, neither the experimental nor the control group in the Spanish sample showed significant differences after intervention. On the contrary, in the Mexican sample, the experimental group showed differences between pre- and postintervention scores, but not the control group (see Supplementary Table 1). Although the overall pattern was for the experimental group to have higher LSI-A scores after the intervention, it only reached statistical significance for the Mexican sample, which showed a stronger effect size. In summary, in the MCI sample, the reminiscence intervention had a significant positive effect in the Mexican sample, with higher scores after intervention, compared to the control condition; but not in the Spanish sample, where there was the same trend, but did not reach statistical significance.

**Fig. 1:**
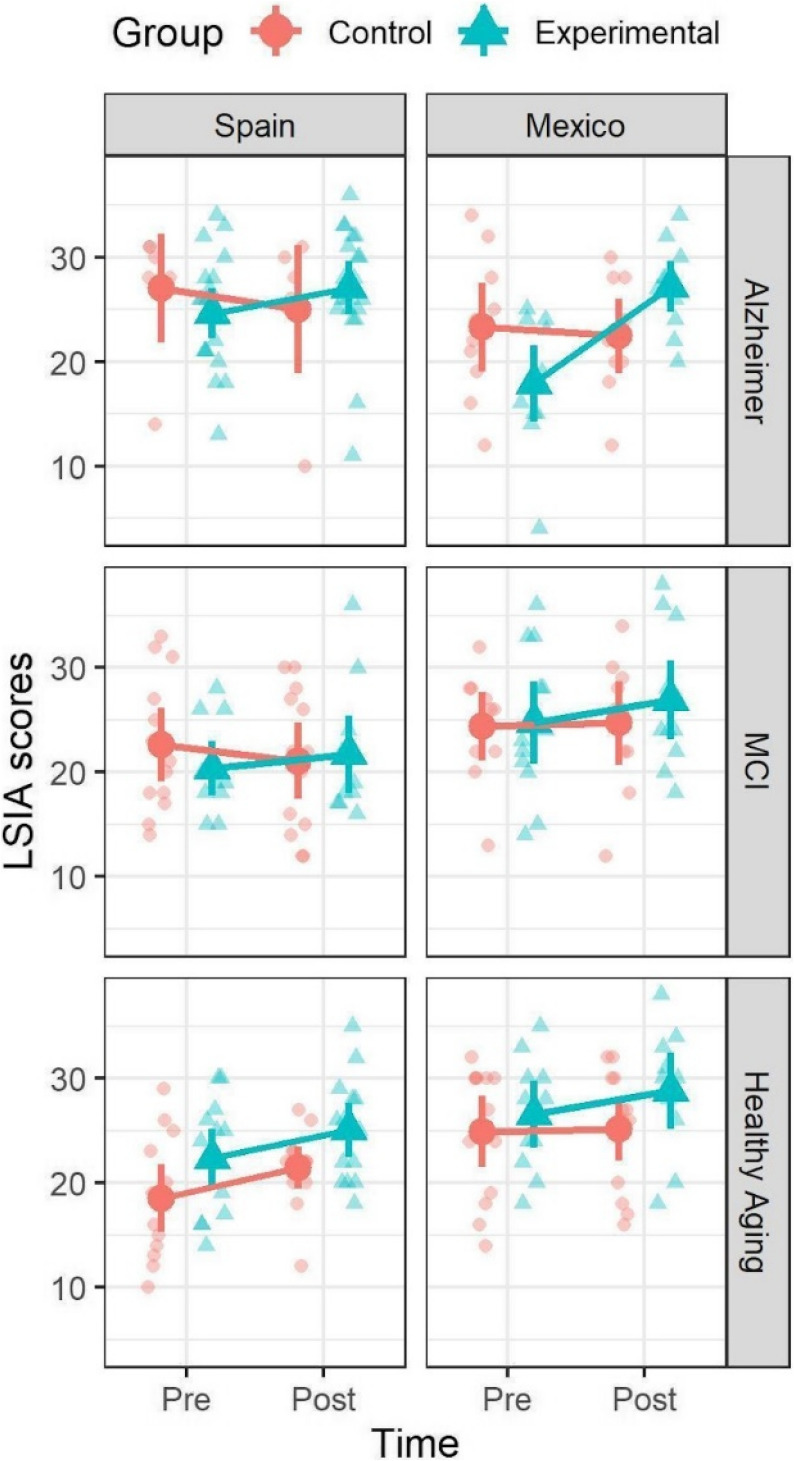
LSIA scores for each type of aging, condition, and country

For the LSI-A scores in the healthy aging sample, neither the triple interaction between time, condition, and country (*t* =  − 0.93, *p* = 0.35) nor the double interaction between time and condition (*t* = 1.8, *p* = 0.076) were significant in the pooled model (see Table 3). In the models for each country, neither showed a significant interaction between time and condition, indicating that the REMPOS intervention did not differ from the control condition in neither sample. Although the hypotheses were that significant differences would appear in all subgroups, none of the tests in the simple main effects analyses reached statistical significance in either the experimental or control group of either country (see Supplementary Table 1). In summary, the overall trend of the interventions in the healthy aging sample was that there were higher LSI-A scores after intervention (i.e., the marginal mean for post intervention scores was higher than preintervention scores), but there was insufficient statistical power to separate the effects in each subgroup Figure 1.

Overall, for LSI-A scores, the Alzheimer’s and MCI Mexican samples showed greater benefits from the reminiscence intervention, compared to the control condition. While the Spanish sample showed some trends of these kinds of benefits, it failed to reach statistical significance. In the healthy aging Spanish and Mexican samples, the overall trend was for better scores after intervention, for both experimental and control conditions, but none reached statistical significance.

### Cross-cultural differences in the effect of the reminiscence program positive specific memories

**Fig. 2:**
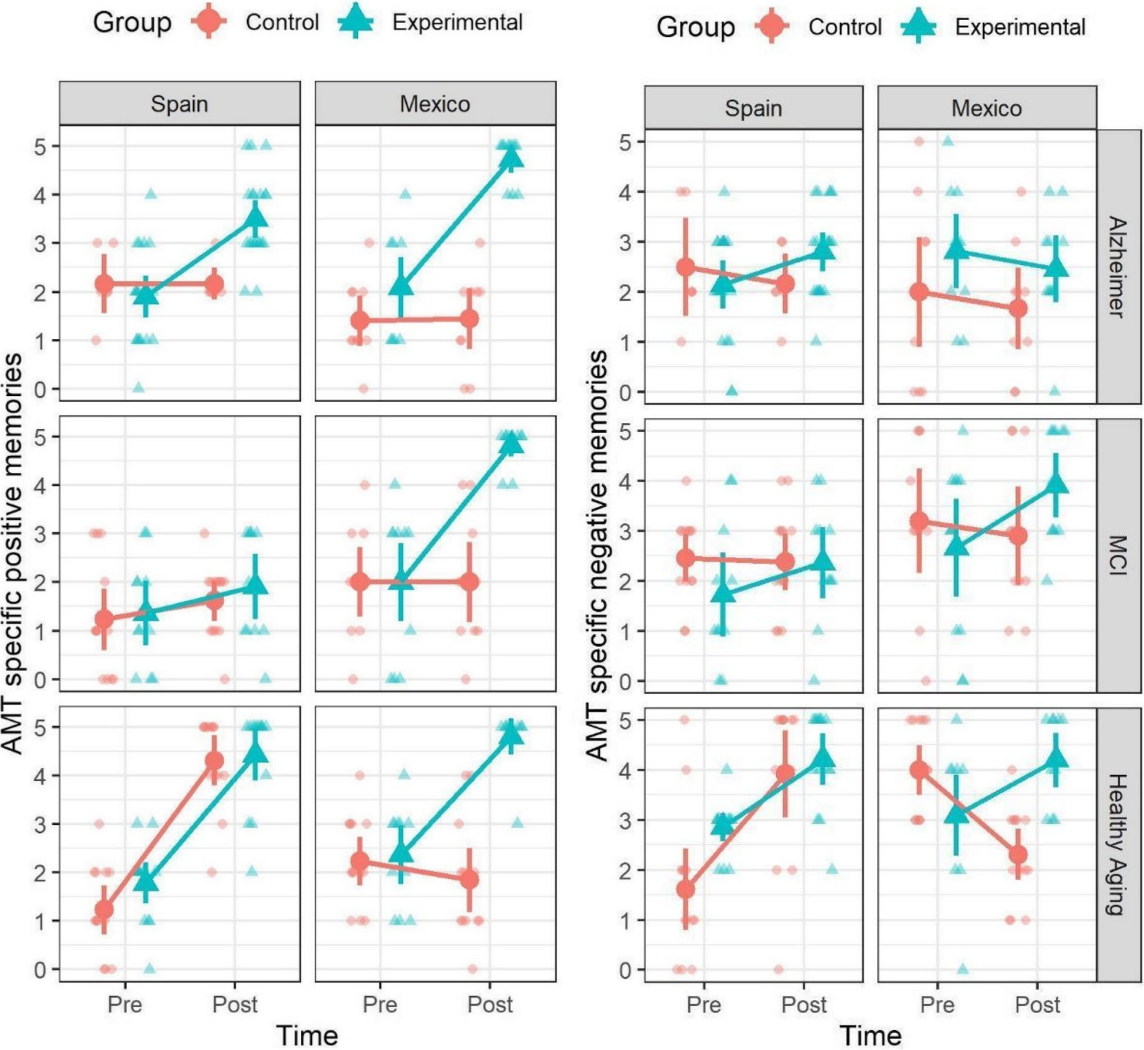
AMT specific positive and negative memories scores for each type of aging, condition, and country

Figure 2 shows the scores of the AMT, both positive and negative specific memories recall. We report the effects of the REMPOS intervention on the AMT-positive specific memories scores with the same structure as the LSI-A scores: the coefficient of interest is the interaction between time and condition, and we report separate models for each country and type of aging.

For the positive specific memories in the Alzheimer’s sample, the triple interaction between predictors time, condition, and country (*t* =  − 3.66, *p* < 0.001) and the double interaction between time and condition (*t* = 8.55, *p* < 0.001) were significant in the pooled model (see Table 3). The double interaction between time and condition was also significant in each country, where the Mexican sample showed the larger coefficient for this interaction. This pattern of results suggests that the experimental group differed from the control group on the effectiveness of the intervention in both countries, but the effect size was larger in the Mexican sample. In the simple main effects analyses, both experimental groups in the Spanish and Mexican samples showed differences between pre- and postintervention scores, where scores increased after intervention. Neither of the control groups showed significant differences (see Supplementary Table 2). In summary, the reminiscence program increased the recall of positive specific memories after intervention, while the control intervention had no effect on this, in both Spanish and Mexican participants with Alzheimer’s disease.

For the positive specific memories in the MCI sample, the triple interaction between predictors time, condition, and country (*t* =  − 5.97, *p* < 0.001) and the double interaction between time and condition (*t* = 7.32, *p* < 0.001) were significant in the pooled model (see Table 3), meaning that the interaction between time and condition, and therefore the differential effect of both types of interventions, varied across countries. In the models for each country, the double interaction between time and condition was only significant in the Mexican sample, and not in the Spanish sample. Simple main effects analyses showed that in the Spanish sample, the experimental and control conditions showed no significant differences between pre- and postintervention scores. In the Mexican sample, the experimental group showed significant differences, but the control group did not (see Supplementary Table 2). In summary, in the MCI sample, the reminiscence program had positive effects increasing recall of specific positive memories in the Mexican, but not in the Spanish sample. The control intervention did not have any effect on the recall of positive specific memories.

For the positive specific memories in the healthy aging sample, the triple interaction between predictors condition, time, and country (*t* =  − 5.41, *p* < 0.001) and the double interaction between time and condition (*t* = 7.46, *p* < 0.001) were significant in the pooled model (see Table 3). Similar to what happened in the MCI sample, the double interaction between time and condition was only significant in the Mexican sample. In the Spanish sample, there was a significant effect of time, indicating that both experimental and control groups improved after the intervention. Simple main effects analyses showed that both experimental and control groups had significantly higher scores after intervention in the Spanish sample. On the other hand, in the Mexican sample, only the experimental group showed significant differences, also with higher post intervention scores (see Supplementary Table 2). In summary, for the healthy aging sample, both interventions had similar positive effects in the Spanish sample, increasing the recall of positive specific memories after intervention, while the reminiscence program had a greater effect than the control condition in the Mexican sample.

Overall, the effect of the reminiscence program compared to the control condition varied across countries in the recall of positive specific memories. In general, the reminiscence program had a positive effect in increasing the recall of positive specific memories in all three types of aging, in both countries, except for the Spanish MCI sample, in which it had no effect. The control condition improved the recall of positive specific memories only in the healthy aging Spanish sample but had no effect in the remaining groups.

### Cross-cultural differences in the effect of the reminiscence program on negative specific memories

For the negative specific memories in the Alzheimer’s sample, neither the triple interaction between condition, time, and country (*t* = 1.46, *p* = 0.148) nor the double interaction between time and condition (*t* = 0.93, *p* = 0.356) were significant in the pooled model (see Table 3). In the models for each country, neither the double interaction between time and condition nor the effects of time were significant. In summary, neither of the reminiscence program and control intervention had any significant effect on recall of negative specific memories in the Alzheimer’s sample.

For the negative specific memories in the MCI sample, the triple interaction between predictors condition, time, and country (*t* =  − 2.24, *p* = 0.028) and the double interaction between time and condition (*t* = 2.84, *p* = 0.006) were significant in the pooled model (see Table 3). Nevertheless, in the models for each country, neither the interaction between time and condition nor the main effects of time were significant. To further analyze this, we ran simple main effects of the predictor time within each condition in each country, but neither of the experimental or control groups in either Spanish or Mexican sample showed significant differences between pre- and postintervention scores (see Supplementary Table 3). Nevertheless, the experimental group in the Mexican sample showed a higher effect size, even when the adjusted *p* value did not reach significance. In summary, the effect of the reminiscence program on the recall of negative specific memories had a small effect in both countries, only detectable in the model that pooled participants from both countries, therefore, with higher statistical power, but not in simple main effects analyses which used adjusted *p* values for multiple comparisons.

For the negative specific memories in healthy aging, the triple interaction between time, condition, and country (*t* =  − 2.74, *p* = 0.008) and the double interaction between time and condition (*t* = 4.23, *p* < 0.001) were significant in the pooled model, indicating that the differences in the effects of the experimental and control conditions varied across countries. When analyzing the models for each country, the double interaction between time and condition was significant only in the Mexican sample. In the simple main effects analyses, both experimental and control conditions showed significant effects in the Spanish sample, with higher recall of negative specific memories after intervention. Interestingly, in the Mexican sample, the control condition showed significant differences between pre- and postintervention scores, where the recall of negative specific memories was lower after intervention, while the experimental condition did not show statistical differences (see Supplementary Table 3). In summary, in healthy aging, the reminiscence program increased recall of negative specific memories in the Spanish sample, but not in the Mexican sample. In the Spanish sample, the control condition also increased recall of negative specific memories, but in the Mexican sample, it showed the opposite effect, decreasing them.

In the case of recall of negative specific memories, the differential effects of reminiscence intervention compared to the control condition varied across countries and types of aging. In the Alzheimer’s and MCI samples, the effects of both interventions were negligible, especially in the Alzheimer’s groups. Interestingly, in the healthy aging sample, there was an unexpected difference across countries: both interventions increased the recall of specific negative memories in the Spanish sample, while the Mexican control condition decreased the recall of those types of memories.

## Discussion

This study presented cross-cultural comparisons of the effectiveness of a reminiscence program against a control group across Mexican and Spanish samples of three types of aging. The results show transcultural considerations of the adaptation of the original Spanish version of the program into a version for Mexican cultural characteristics [42].

The effects on life satisfaction were similar across countries. In the experimental groups (AD, MCI and HA) for both countries, there was an increase in the LSI-A scores after the intervention, while in the control groups (AD, MCI and HA), either the scores were maintained, or they tended to decrease. Nevertheless, this increase in scores was only significant in Mexico’s experimental group for the AD and MCI type of aging in comparison to Spain’s.

For the AMT scores, we found that the recall of specific positive memories increased after the intervention, especially for the experimental groups in all types of aging (AD, MCI, and HA) for both countries, except for the Spanish MCI group. Specifically, we observed a statistically significant increase of specific positive memories scored for both countries’ experimental groups, while the opposite pattern was observed for the control groups, with the exception of Spain’s HA control group in which the scores were higher after the intervention.

As for the recall of specific negative memories, no significant differences were found throughout the groups, with the exception of Spain’s HA groups (both control and experimental) and the Mexican HA control group, which showed a significant increase and a decrease, respectively, in recall of specific negative memories. Both significant results are opposite to the expected effects because we expected that by having a space in which to share and express reminiscences, these would tend to decrease. Although reminiscing was considered as a possible sign of dysfunction or cognitive impairment in older age, it is currently considered to have adaptive functions serving as a positive predictor of mental health in older people [43]. Interventions based in reminiscence therapy are associated with a significant increase of general cognitive function, a decrease in depressive symptomatology, an increase in life satisfaction and a better recall of specific positive and negative memories [22, 44].

In previous research [22, 44], where REMPOS therapy was applied to autonomous older people with different levels of cognitive impairment, the experimental group showed a statistically significant increase in cognitive function, life satisfaction, and self-esteem, as well as a decrease in depressive symptoms compared to the control group. In this research, we extended the previous data to test the effectiveness of the REMPOS program cross-culturally in Mexico and Spain, also considering the normal and pathological aging factors that were previously mentioned.

The improvement of the quality of life should be one of the main objectives when working with older people. In Europe, the economic growth has positively impacted older people’s quality of life. On the contrary, Mexico’s economic growth and development is barely on the rise [45]. Considering both social and economic differences across countries, the comparison of the effects on life satisfaction and autobiographical memories of the positive reminiscence program is of interest.

The usefulness of the results found is that using this type of intervention strategy and taking the previously mentioned results as a reference, the life satisfaction of the older adults who were part of the experimental groups in the Mexican sample is increased, especially with respect to all of those who scored low prior to the intervention. In addition, after the intervention, we found an increase in specific positive memories in both countries, and this effect is greater in the experimental group in patients with AD. On the other hand, recent studies [46–50] have shown that reminiscence intervention can be effective in the improvement of depression, as well as different emotional and personal variables related to the well-being and quality of life of the elderly.

This study explored how a positive reminiscence (REMPOS) therapy relates to cognitive (autobiographical memory) and affective variables (life satisfaction) in older people. Significant improvements stand out between the pretest and posttest scores in relation to the intervention (experimental and controls) in the experimental groups (AD, MCI, and HA) for both countries. In conclusion, this study has shown positive results using a non-pharmacological therapy in older people. People with cognitive impairment (specifically AD) showed a better improvement in cognition and emotional aspects. Thus, the results are quite encouraging and indicate the need to continue promoting studies on this topic since the use of REMPOS had shown to significantly decrease depressive symptoms, improve cognition and life satisfaction, and increase the recall of specific positive memories, which are all imperative factors in the psychological well-being and quality of life of older people. The intervention studied here has had non-commercial interests and requires further studies to replicate and implement with more diverse populations, for which the materials of the REMPOS program are available upon request to any institutions that may need it.

### Limitations

Although both countries share similarities in culture, there are  some differences that we consider relevant to have a better control for future research. First, there were more women than men participants, with 58 females and 19 males for the Spanish sample and 51 females and 16 males for the Mexican sample. Having a more balanced sample between males and females could improve the clarity of the results shown. Second, the education level was lower in the Mexican sample than the Spanish sample; this is due to cultural and historical differences between both countries as most older people in Mexico tend to have only elementary or secondary education level. Third, not all the instruments used in this study have been validated for Mexico, here we come across the methodological and cross-cultural challenges of psychology today since most of the instruments used in psychological evaluation have been created and validated within Anglo-Saxon western societies and may show a cultural bias when used in other populations. Fourth, regarding the duration of the REMPOS intervention, we still do not know if more sessions could mean a more positive outcome or not. Doing more research of the time of sessions, frequency, and total duration could help improve the efficiency of the REMPOS program.

### Supplementary Information

**Additional file 1: Supplementary Table 1. **

**Additional file 2: Supplementary Table 2. **

**Additional file 3: Supplementary Table 3. **

## Data Availability

The datasets used and/or analyzed during the current study are available from the corresponding author on reasonable request.
