# Supplementary material for: Cross-cultural effects of reminiscence therapy on life satisfaction and autobiographical memory of older adults: a pilot study across Mexico and Spain
Source: Alzheimers Res Ther. 2023 Nov 22;15:204. doi: 10.1186/s13195-023-01347-x (PMC10664501; doi:10.1186/s13195-023-01347-x)
Supplement: Supplementary file 1 — Additional file 1: Supplementary Table 1. [file 13195_2023_1347_MOESM1_ESM.docx]

**Supplementary Table 1.**

Comparison between pre and post intervention scores in LSIA.

| **LSIA** | n | Pre  Mean (SD) | Post  Mean (SD) | t | df | p | Hedge’s g |
| --- | --- | --- | --- | --- | --- | --- | --- |
| **Alzheimer** |  |  |  |  |  |  |  |
| **Spain** |  |  |  |  |  |  |  |
| Experimental | 20 | 24.6 (5.5) | 27.0 (5.8) | -2.02 | 19 | .172 | -0.43 |
| Control | 6 | 27.0 (6.5) | 25.0 (7.7) | 1.65 | 5 | .320 | 0.57 |
| **Mexico** |  |  |  |  |  |  |  |
| Experimental | 11 | 17.9 (6.2) | 27.2 (4.1) | -5.40 | 10 | .001 | -1.50 |
| Control | 9 | 23.3 (6.9) | 22.4 (5.7) | 0.71 | 8 | .496 | 0.22 |
| **MCI** |  |  |  |  |  |  |  |
| **Spain** |  |  |  |  |  |  |  |
| Experimental | 11 | 20.4 (4.4) | 21.7 (6.3) | -1.05 | 10 | .897 | -0.29 |
| Control | 13 | 22.6 (6.5) | 21.1 (6.8) | 1.09 | 12 | .897 | 0.28 |
| **Mexico** |  |  |  |  |  |  |  |
| Experimental | 11 | 24.8 (7.0) | 26.9 (6.7) | -3.68 | 10 | .017 | -1.02 |
| Control | 10 | 24.4 (5.3) | 24.7 (6.4) | -0.18 | 9 | .897 | -0.05 |
| **Healthy aging** |  |  |  |  |  |  |  |
| **Spain** |  |  |  |  |  |  |  |
| Experimental | 14 | 22.4 (5.3) | 25.1 (5.0) | -1.32 | 13 | .416 | -0.33 |
| Control | 13 | 18.5 (5.9) | 21.5 (3.7) | -2.32 | 12 | -154 | -0.60 |
| **Mexico** |  |  |  |  |  |  |  |
| Experimental | 10 | 26.5 (5.4) | 28.8 (6.1) | -1.73 | 9 | .357 | -0.50 |
| Control | 13 | 24.9 (6.2) | 25.2 (5.6) | -0.13 | 12 | .897 | -0.03 |
