# Supplementary material for: Cross-cultural effects of reminiscence therapy on life satisfaction and autobiographical memory of older adults: a pilot study across Mexico and Spain
Source: Alzheimers Res Ther. 2023 Nov 22;15:204. doi: 10.1186/s13195-023-01347-x (PMC10664501; doi:10.1186/s13195-023-01347-x)
Supplement: Supplementary file 2 — Additional file 2: Supplementary Table 2. [file 13195_2023_1347_MOESM2_ESM.docx]

**Supplementary Table 2.**

Comparison between pre and post intervention scores in AMT positive specific memories.

| **AMT positive specific memories** | n | Pre  Mean (SD) | Post  Mean (SD) | t | df | p | Hedge’s g |
| --- | --- | --- | --- | --- | --- | --- | --- |
| **Alzheimer** |  |  |  |  |  |  |  |
| **Spain** |  |  |  |  |  |  |  |
| Experimental | 20 | 1.9 (1.0) | 3.5 (0.9) | -6.0 | 19 | <.001 | -1.29 |
| Control | 6 | 2.2 (0.8) | 2.2 (0.4) | 0 | 5 | 1.00 | 0.00 |
| **Mexico** |  |  |  |  |  |  |  |
| Experimental | 11 | 2.1 (1.0) | 4.7 (0.5) | -9.46 | 10 | <.001 | -2.63 |
| Control | 9 | 1.4 (0.8) | 1.4 (1.0) | 0.29 | 8 | 1.00 | 0.09 |
| **MCI** |  |  |  |  |  |  |  |
| **Spain** |  |  |  |  |  |  |  |
| Experimental | 11 | 1.4 (1.1) | 1.9 (1.1) | -1.07 | 10 | .933 | -0.30 |
| Control | 13 | 1.2 (1.2) | 1.6 (0.8) | -0.96 | 12 | .933 | -0.25 |
| **Mexico** |  |  |  |  |  |  |  |
| Experimental | 11 | 2.0 (1.4) | 4.8 (0.4) | -6.10 | 10 | <.001 | -1.70 |
| Control | 10 | 2.0 (1.2) | 2.0 (1.3) | 0 | 9 | 1.00 | 0.00 |
| **Healthy aging** |  |  |  |  |  |  |  |
| **Spain** |  |  |  |  |  |  |  |
| Experimental | 14 | 1.8 (0.8) | 4.4 (1.0) | -11.7 | 13 | <.001 | -2.95 |
| Control | 13 | 1.2 (0.9) | 4.3 (0.9) | -9.34 | 12 | <.001 | -2.43 |
| **Mexico** |  |  |  |  |  |  |  |
| Experimental | 10 | 2.4 (1.0) | 4.8 (0.6) | -5.81 | 9 | <.001 | -1.68 |
| Control | 13 | 2.2 (0.9) | 1.9 (1.2) | 1.10 | 12 | .293 | 0.29 |
