# Supplementary material for: Cross-cultural effects of reminiscence therapy on life satisfaction and autobiographical memory of older adults: a pilot study across Mexico and Spain
Source: Alzheimers Res Ther. 2023 Nov 22;15:204. doi: 10.1186/s13195-023-01347-x (PMC10664501; doi:10.1186/s13195-023-01347-x)
Supplement: Supplementary file 3 — Additional file 3: Supplementary Table 3. [file 13195_2023_1347_MOESM3_ESM.docx]

**Supplementary Table 3.**

Comparison between pre and post intervention scores in AMT negative specific memories.

| **AMT negative specific memories** | n | Pre  Mean (SD) | Post  Mean (SD) | t | df | p | Hedge’s g |
| --- | --- | --- | --- | --- | --- | --- | --- |
| **Alzheimer** |  |  |  |  |  |  |  |
| **Spain** |  |  |  |  |  |  |  |
| Experimental | 20 | 2.2 (1.1) | 2.8 (0.9) | -2.94 | 19 | .034 | -0.63 |
| Control | 6 | 2.5 (1.2) | 2.2 (0.8) | 0.44 | 5 | .906 | 0.15 |
| **Mexico** |  |  |  |  |  |  |  |
| Experimental | 11 | 2.8 (1.3) | 2.5 (1.1) | 0.89 | 10 | .906 | 0.25 |
| Control | 9 | 2.0 (1.8) | 1.7 (1.3) | 1.10 | 8 | .906 | 0.33 |
| **MCI** |  |  |  |  |  |  |  |
| **Spain** |  |  |  |  |  |  |  |
| Experimental | 11 | 1.7 (1.4) | 2.4 (1.2) | -1.25 | 10 | .720 | -0.35 |
| Control | 13 | 2.5 (0.9) | 2.4 (1.0) | 0.25 | 12 | 1.00 | 0.06 |
| **Mexico** |  |  |  |  |  |  |  |
| Experimental | 11 | 2.7 (1.7) | 3.9 (1.1) | -2.61 | 10 | .104 | -0.73 |
| Control | 10 | 3.2 (1.7) | 2.9 (1.6) | 0.49 | 9 | 1.00 | 0.14 |
| **Healthy aging** |  |  |  |  |  |  |  |
| **Spain** |  |  |  |  |  |  |  |
| Experimental | 14 | 2.9 (0.5) | 4.2 (1.0) | -5.47 | 13 | <.001 | -1.38 |
| Control | 13 | 1.6 (1.5) | 3.9 (1.6) | -3.89 | 12 | .004 | -1.01 |
| **Mexico** |  |  |  |  |  |  |  |
| Experimental | 10 | 3.1 (1.4) | 4.2 (0.9) | -1.63 | 9 | .137 | -0.47 |
| Control | 13 | 4.0 (0.9) | 2.3 (0.9) | 7.14 | 12 | <.001 | 1.85 |
